# Characteristics of adenine nucleotides content in acute hypoxia by mathematical analysis methods

**DOI:** 10.1186/2193-1801-4-S1-P11

**Published:** 2015-06-12

**Authors:** Elena Erlykina, Albina Moshkova

**Affiliations:** Nizhny Novgorod State Medical Academy, Russian Federation; Nizhny Novgorod State Technical University n.a. R.E. Alekseev, Russian Federation

**Keywords:** adenylate pool, hypoxia, multiregression model

Oxygen starvation is observed in a variety of pathological states and serves as one of the urgent problems in medicine. A decrease in oxygen supply to tissues is accompanied by the inhibition of metabolic processes (primarily of energy metabolism), which impairs functional activity of the brain. The main source of energy for brain is adenosine triphosphate (ATP). It was shown that the components of adenylate pool can be used as early predictors of hypoxia. Aim of the study: the aim of our study was analysis of adenosine triphosphate (ATP) and adenosine monophosphate (AMP) experimental concentrations and integral coefficient K= in intact animals brain tissue and in disturbances of the oxygen regime by methods of mathematical analysis, as well as detection of some regularity in the character of their changes under the impact of hypoxia for the assessment and prediction of direction of production and utilization energy in metabolic pathways. Methodology: in this study empirical dependencies and criteria of statistical significance of mathematical modeling of quantitative relation between specified brain nucleotide stock indicators for the assessment and prognostication of brain energy state in extreme conditions were used. Results and area of their application: The use of empirical dependencies methods allowed to create multiregression models, subtly enough to unite experimental indicators ATP and AMP in hypobaric hypoxia and ischemia with different-term exposure. Obtained models can be used for prognostication of ATP and AMP concentrations in disturbances of the oxygen regime in a short or over a long period of time, as well as to receive information of indicator K= changing depending on brain hypoxia. Conclusion: functional dependencies are presented in this study to analyze shape, closeness and stability of relations between adenine nucleotides characterizing coupling of production and utilization energy processes, and also to predict the direction of these processes under hypoxic condition.

